# A cost-effectiveness analysis of two psychological treatments for controlled drinking in individuals with alcohol use disorder

**DOI:** 10.1186/s12962-025-00633-9

**Published:** 2025-06-02

**Authors:** Sergio Flores, Egill Jónsson-Bachmann, Stina Ingesson-Hammarberg, Anders Hammarberg, Camilla Nystrand, Filipa Sampaio

**Affiliations:** 1 Department of Public Health and Caring Sciences (IFV), Uppsala University, BMC, Husargatan 3, Uppsala, 751 22 Sweden; 2 Centre for Psychiatry Research, Department of Clinical Neuroscience, Karolinska Institutet, & Stockholm Health Care Services, Region Stockholm, Stockholm, Sweden; 3 Department of Learning, Informatics, Management and Ethics, Karolinska Institute, Stockholm, Sweden

**Keywords:** Alcohol use disorder, Behavioral self-control training, Motivational enhancement therapy, Cost analysis, Cost-effectiveness

## Abstract

**Background and aims:**

In Sweden, up to 7% of people exhibit harmful alcohol use or dependency, but only a small fraction (10–20%) pursue treatment. While Behavioral Self-Control Training (BSCT) and Motivational Enhancement Therapy (MET) are effective for alcohol use disorders (AUD), their cost-effectiveness has not been studied. This research evaluates the cost-effectiveness of BSCT and MET in treating AUD, focusing on achieving controlled drinking from both a healthcare and limited societal perspective.

**Design, setting, and participants:**

A Markov model was created to compare the cost-effectiveness of BSCT and MET for treatment-seeking individuals with AUD. This model simulated a sample of patients with AUD over five years, calculating the costs and outcomes for both treatments. It used a cost-utility analysis to determine the cost per quality-adjusted life year (QALY). The model accounted for varying levels of alcohol intake and related health complications. Efficacy data came from a randomized trial, with complication risks, costs, utilities, and mortality rates drawn from existing literature.

**Intervention and comparator:**

BSCT, consisting of five sessions, was compared to MET, constituted by four sessions.

**Measurements:**

The primary outcome was cost per QALY. Probabilistic and univariate sensitivity analyses were performed to estimate how parameter uncertainty affected model outputs.

**Findings:**

BSCT proved less costly than MET, yielding savings in healthcare costs by $50.52 and societal costs by $118.12 on average per person. Additionally, BSCT demonstrated an advantage in QALYs, with a gain of 0.060 QALYs per patient over MET. Sensitivity analyses confirmed the robustness of these results, indicating that BSCT is the cost-effective treatment option.

**Conclusions:**

In comparison to MET, BSCT is less costly and more beneficial for health, indicating its potential as a valuable treatment option for individuals with AUD targeting controlled drinking. The model can be applied to assess the cost-effectiveness of various other AUD treatment interventions.

**Supplementary Information:**

The online version contains supplementary material available at 10.1186/s12962-025-00633-9.

## Introduction

Harmful use of alcohol is one of the leading contributors to disease and premature death globally [1]. Up to 7% of the Swedish population meets the criteria for harmful use or alcohol dependency according to the International Classification of Diseases-10th version (ICD-10) criteria [2]. However, only 10–20% of individuals with AUD seek treatment [3]. Many of these ultimately reject or interrupt treatment when the goal of treatment is total abstinence from alcohol consumption [4, 5]. Conversely, treatment uptake may increase when offered with controlled drinking as an optional treatment goal [6, 7]. Therefore, providing treatments suitable for both abstinence- and controlled-drinking goals may contribute to an increase in treatment initiation and retention, and positive treatment outcomes [8–12].

The risk of developing alcohol-induced morbidities for alcohol consumers increases substantially with consumption over a longer time period. For example, studies have demonstrated that the risk of diseases such as cancer, chronic liver diseases, neuropsychiatric disorders, cardiovascular diseases, and unintentional death increases significantly with prolonged harmful drinking patterns [13, 14]. Furthermore, the risk increases linearly as consumption levels rise [13]. Accordingly, minimizing harmful consumption levels is fundamental in reducing short- and long-term consequences and morbidities in AUD.

Moreover, the consequences of high consumption levels lead to severe economic ramifications. For instance, annual gross costs of alcohol consumption in Sweden have been estimated at 0,9% of the Gross Domestic Product, an annual societal cost per adult with alcohol dependency of $US 246 (2019 value). A significant proportion of costs is attributable to productivity losses resulting from work absence, short- and long-term sick leave, early retirement, and mortality [15]. Additionally, annual net health-related losses have been estimated at 121,800 quality-adjusted life years (QALYs), and 27,962 potential life years [16].

It is fundamental to estimate cost-effectiveness by focusing on health losses and healthcare costs related to the short- and long-term effects of alcohol-induced morbidities in order to capture the broad impact of alcohol use disorders (AUDs). This approach ensures that economic evaluations of treatments aimed at reducing alcohol consumption levels take into account the wide-ranging and long-term consequences of alcohol consumption [1, 17]. To a proportion of individuals with the disorder, alcohol use disorder (AUD) can be a chronic disease involving recurring cycles of harmful consumption patterns, and relapse [1, 17]. Recommended treatments for AUD should therefore aim to reduce health-related risks of alcohol consumption, which in turn should minimize long-term costs to society.

Controlled drinking is defined as when individuals with a previous drinking pattern of alcohol at harmful levels, returns to a stable low-risk consumption pattern [18]. One psychological treatment method aimed for controlled drinking is Behavioral Self-Control Training (BSCT). BSCT is a treatment based on Cognitive Behavioral Therapy (CBT), with a specific focus on skills training based on the patient’s own goal. BSCT was evaluated mainly during the 1970s and 80s [19–22]. Unfortunately, there is a lack of studies examining the outcome of BSCT compared with other treatments with the goal of controlled drinking [18, 23, 24], and the few available meta-analyses and reviews have shown no differences in treatment outcomes for BSCT compared with abstinence-oriented treatments [25, 26]. Motivational Enhancement Therapy (MET), however, has shown promise in several trials despite its short format [27, 28] and is a fitting benchmark for BSCT because MET can be effectively used to support individuals aiming for controlled drinking. According to the Swedish National Board of Health and Welfare’s 2019 guidelines, MET is a preferred AUD treatment due to its cost-effectiveness compared to alternatives like Social Behavioral Network Therapy and others [29–31].

Results have demonstrated MET’s cost-effectiveness in some settings, although the evidence’s strength is unconvincing. For instance, the comparators in previous evaluations have either been treatments aiming for complete abstinence [30], or a brief intervention which may not be comparable to a longer treatment intervention [31]. More importantly, these economic evaluations were limited to the relatively short follow-up time (6 to 12 months) of the trials they were based on. Consequently, the evaluations were not able to analyze the longer-term cost-effectiveness by including alcohol-induced morbidities and costs related to different levels of consumption, which hampers the current understanding of the cost-effectiveness of MET. These limitations highlight the importance of the current study, which seeks to fill this knowledge void by evaluating the medium-term cost-effectiveness of BSCT and MET for individuals with AUD, focusing on controlled drinking as the treatment goal.

## Methods

### Analytical approach

The current study adopted a cost-utility analysis framework based on CHEERS best practices recommendations to estimate the costs and benefits, measured using quality-adjusted life years (QALY), of BSCT and MET treatment in individuals diagnosed with AUD. The analysis adopted separately a healthcare and a limited societal perspective. Healthcare costs included costs of procedures necessary for diagnosis, treatment, and follow-up of alcohol use disorder and alcohol-attributable events. Societal costs included productivity losses due to the significant impact of alcohol use disorder and alcohol-related events on absenteeism from work [15].

Efficacy data and patient characteristics were sourced from a randomized controlled trial (RCT) comparing BSCT and MET, conducted at three clinics within the Stockholm Centre for Dependency Disorders in Sweden. The primary clinical outcome results from this RCT, mean weekly alcohol consumption at 26 weeks, indicated no statistically significant difference between BSCT and MET (t = -0.278, *P* = 0.781, d = -0.22, 95% CI = -0.60 to 0.16) [32]. Outcome measures were collected at study inclusion (baseline), 12 weeks, 26 weeks, and 52 weeks.

We modelled a multi-cohort of individuals diagnosed with AUD employing a two-part Markov model – the first part lasting a year with three-month cycles and the second lasting four years with yearlong cycles, and estimated the expected costs and health-related outcomes of delivering both interventions applying a 3% discount rate chosen based on standard practices in health economic evaluations to reflect the time preference for costs and benefits occurring in the future. A five-year time horizon was used in this study in order to fully capture the effects of the interventions without stretching the assumptions for a whole lifetime, an approach which has also been used in other economic evaluations of AUD interventions [17]. This time horizon is used since other factors, such as changes in healthcare policies, advancements in treatment methods, and patient adherence rates, can impact the costs and benefits attributable to an intervention over longer periods of time.

### Participants

Our model simulated 250 individuals diagnosed with AUD according to The Diagnostic and Statistical Manual of Mental Disorders, Fifth Edition (DSM-5), with characteristics derived from the trial participants [32]. A complete list of inclusion and exclusion criteria and a description of the population’s baseline characteristics are in Table 1.

### Intervention description

#### BSCT

Behavioral Self-Control Training (BSCT) is a psychological treatment method for controlled drinking, based on cognitive behavioral therapy (CBT) [33]. In the present study, a Swedish clinicians’ manual was used, which was adapted from the self-help manual by Miller and Muños [34]. In the current study, the BSCT treatment consisted of five individual treatment sessions of 45 min each for a total duration of 12 weeks, including the following components:: Session (1) Feedback on initial assessments, goal setting; and registration of consumption with the help of an alcohol diary; Session (2) Analysis of risk situations for unwanted alcohol consumption (functional analysis); Session (3) Skills to limit alcohol intake in drinking situations (moderation strategies); Session (4) Skills to increase days with abstinence; Session (5) Maintenance plan.

#### Comparator

Motivational Enhancement Therapy (MET) is a semi-structured manual-based treatment with the aim of strengthening the individual’s motivation to change [35, 36]. In the present study, MET consisted of four individual sessions lasting 45 min, during a 12-week period, with the following session structure: 1) feedback on assessment and a Motivational Interviewing (MI) session 2–4) MI sessions. The first session was based on an initial assessment of biological, psychological, and social consequences related to alcohol, followed by a series of sessions with a focus on supporting behavior change. The therapist had the opportunity to use two work sheets: (a) Change plan and (b) Formulation of a plan for long-term maintenance, for further support of the patient’s goals.

#### Fidelity of intervention delivery

On average, participants attended 3.8 BSCT sessions (SD = 1.4) and 3.6 MET sessions (SD = 1.3) from baseline to 12 weeks post inclusion. BSCT sessions lasted 45.1 min, and MET sessions lasted 44.5 min.

**Table 1:** Baseline descriptive and clinical characteristics

		BSCT (*n* = 125)		MET (*n* = 125)	
Gender (% female)		42		54	
		**Mean**	**SD**	**Mean**	**SD**
Number of sessions attended		3.8	1.4	3.6	1.3
Age (years)		52.2	11.4	51.4	10.7
AUD (number of DSM-5 criteria)		5.39	1.93	5.14	2.03
Mean weekly consumption (standard drinks)		23.65	12.44	24.34	12.75
Proportion of heavy drinking days (of the last 30 days)		0.28	0.26	0.31	0.28
		**N**	**%**	**n**	**%**
Educational Status					
	Basic Education	5	4.1	3	2.6
	Upper Secondary School	24	19.5	17	13.8
	Post-secondary school				
	University < 3 years	20	16.3	27	22.0
	University > 3 years	62	50.4	62	50.4
Occupational Status					
	Employed/self-employed	98	79.7	106	86.2
	Retired/housekeeper	18	14.6	9	7.3
	Student/parental leave	1	0.8	2	1.6
	Unemployed/sick leave	6	4.9	6	4.9

Note: AUD = Alcohol use disorder; DSM-5 = Diagnostic and Statistical Manual of Psychiatric Disorders, 5th Edition

### Intervention efficacy

The efficacy of the interventions was measured as change in mean weekly alcohol consumption from baseline to each follow-up timepoint, i.e., 12, 26, and 52 weeks, for the assessment of alcohol consumption according to measurements of Swedish standard drinks, using clinician based interviews with The Timeline Follow back Interview Method of 90 days [37]. Reported standard drinks were converted to grams, where each drink corresponded to 12 g. Daily consumption in grams was then averaged across the last 90 days. These average estimates were categorized according to the World Health Organization’s (WHO) Drinking Risk Levels (DRLs), adjusted for sex [38].

### Determining drinking risk levels

The average daily consumption of pure alcohol in grams in participants included in the trial was categorized into DRLs using the *International guide for monitoring alcohol consumption and related harm* [38] by the WHO. Each DRL was associated with a risk for temporary and medium-term health complications for men and women, as seen in Table 2.

**Table 2:** Categorical levels of complications related to the average volume of pure alcohol per day for women and men according to WHO drinking risk levels

Category	Average volume of pure alcohol per day for women (g)	Average volume of pure alcohol per day for men (g)
**Temporary complications**		
Low Risk	0–20	0–40
Medium Risk	21–40	41–60
High Risk	41–60	61–100
Very High Risk	> 61	> 101
**Medium term complications**		
Low Risk	0–20	0–40
Medium Risk	21–40	41–60
High Risk	≥ 41	≥ 61

Note: WHO = World Health Organization

### Calculating incidence of alcohol-related harmful events and death

Risk Equations, developed by the Canadian Centre for Addiction and Mental Health (CAMH) [39], were used to translate DRLs into personal risks of complications for the trial participants. The following equation was used:

$$ \begin{array}{l}{\rm{Personal}}\,{\rm{Risk}}\,{\rm{Event}}\,\left( {{\rm{i,}}\,{\rm{x}}} \right)\,{\rm{ = }}\,{\rm{Population}}\,{\rm{Risk}}\,{\rm{Event}}\,\left( {\rm{i}} \right)\, \times \,\\{\rm{Relative}}\,{\rm{Risk}}\,{\rm{Event}}\,\left( {\rm{i}} \right)\left( {\rm{x}} \right)\end{array} $$

Population Risk Events for the alcohol-related complications, outlined in Table 2, were defined as the population incidence in 2021. These were obtained from the Diagnosis Database held by the Swedish National Board of Health and Welfare Statistical Database using their respective ICD-10 diagnosis [40, 41]. The relative risk for the alcohol-related complications were sourced from both an updated meta-analysis by Rehm et al. [42] and a risk of injury analysis by Cherpitel et al. [43]. Personal risk event estimates were transformed into mortality and morbidity probabilities for each alcohol-related event by sex. These are shown in Tables A1-A4 in the appendix.

### Model structure

**Fig. 1:**
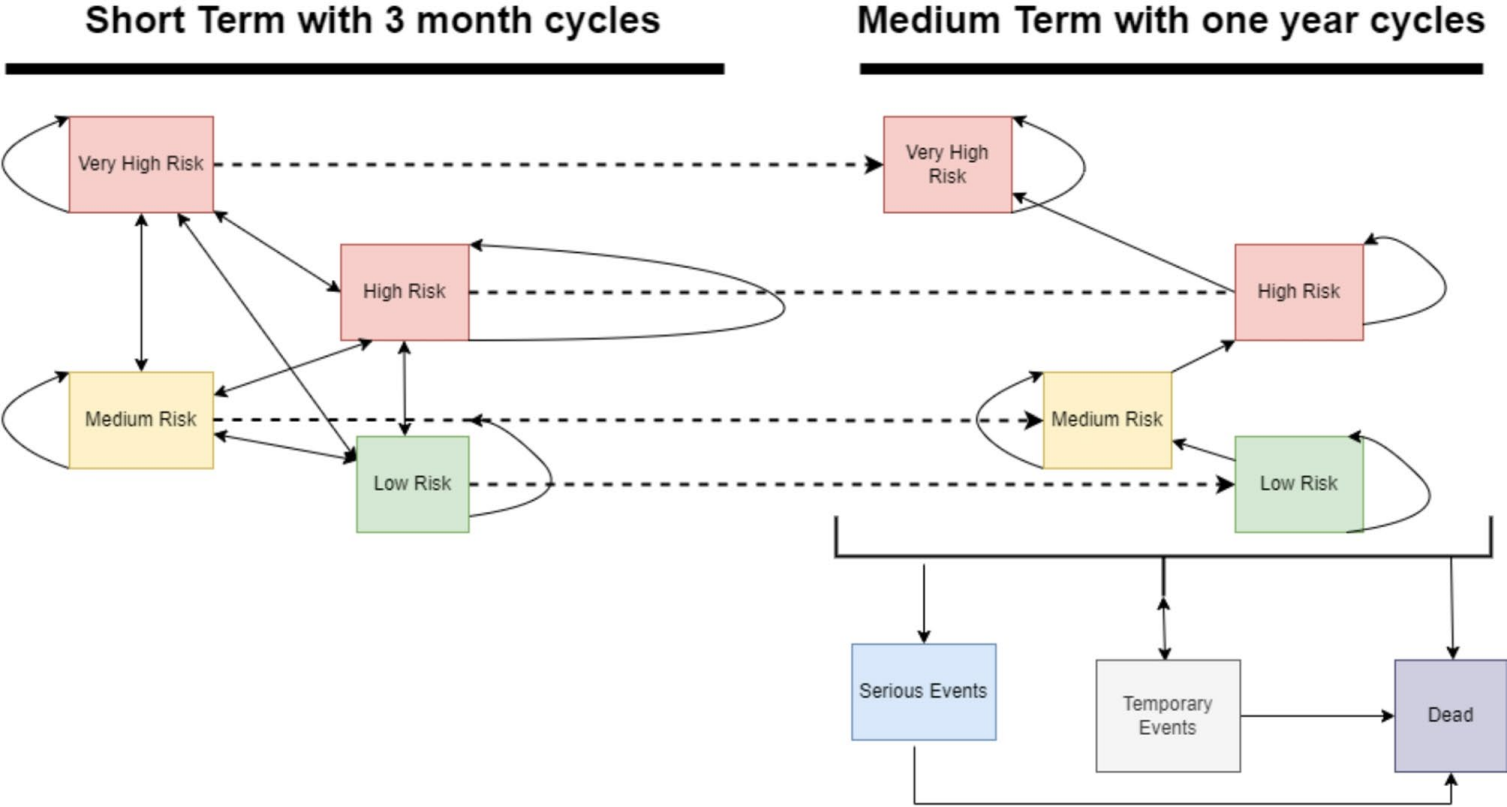
Model structure

A Markov model was selected as the best option to model AUD since individuals with AUD can transition between different levels of risk drinking based on well-documented estimates from WHO [38]. Figure 1 depicts the conceptual model structure used in this study.

The model is divided into two temporal parts: a short-term phase and a medium-term phase. Throughout the model, participants move through four key health states according to DRLs stated by the WHO: very high, high, medium, and low.

A three-month cycle length was applied to match the trial measurement time points during the short-term phase, lasting a total of one year. Individuals entered the short-term phase of the model at different DRL levels depending on the consumption pattern for each patient, 90 days prior to the start of the trial. In each cycle, individuals either transitioned between any of the four short-term DRL states or remained in the DRL state they were currently in, based on data from the trial.

In the medium-term phase, the cycle length was set to one year. Individuals could transition between the same DRL states as in the short-term part of the model. Probabilities for transitions between DRL states were derived from the trial data [32] for the short-term phase. For the medium-term phase, these transitions were informed by literature; for example, to model relapse to more harmful consumption patterns, an annual probability of 14% was applied. This figure was derived from Taylor et al. (44; Table 1), who reported that 86% of individuals in their cohort maintaining ‘12 months social drinking’ in a given year retained that status into the following year, implying a 14% annual probability of transitioning out of that ‘social drinking’ state. In our model, this 14% annual probability applied to individuals in the ‘Low Risk’ DRL state transitioning to ‘Medium Risk’, and to those in the ‘Medium Risk’ DRL state transitioning to ‘High Risk’, reflecting a one-step worsening from states analogous to Taylor et al.‘s ‘social drinking’. Probabilities for transitions to alcohol-attributable events and death are detailed in Appendix Tables A1-A4 and A7.

Throughout the medium-term phase of the model, each DRL health state was connected to risks for medium-term or temporary alcohol-attributable events, as listed in Table 3. These events were chosen based on the following criteria: (i) alcohol-attributable diseases or injuries that are known to incur a high cost to both healthcare and society, (ii) have shown to be associated with alcohol consumption, and (iii) would occur during the length of the model. Temporary alcohol-related events were modeled as tunnel states lasting the length of a long-term cycle (i.e., one year), while alcohol-related medium-term events meant individuals moved to distinct, absorbing (permanent) health state specific to that event for the remainder of the model horizon, or until death. Once an individual entered such a permanent complication state, they could not experience another listed medium-term event, and their transitions between the original drinking risk level (DRL) states were no longer tracked, with subsequent costs and quality of life primarily determined by the complication. In addition, at any given state within the model, the participants could also move into a death state.

**Table 3:** Alcohol attributable events included in the model

Event	Modeled as
**Medium Term**	
Ischemic heart disease [45]	Permanent State
Ischemic stroke [46, 47]	Permanent State
Hemorrhagic stroke [46, 47]	Permanent State
Cirrhosis of the liver [13, 48]	Permanent State
Chronic Pancreatitis [49]	Permanent State
**Temporary**	
Lower respiratory infections [50]	Tunnel State
Transport injuries [51]	Tunnel State
Injuries other than from transport [52]	Tunnel State

### Model parameters

Five broad categories of parameters were used in the model: (1) costs for AUD treatments and treatment of alcohol-attributable events; (2) utilities for DRLs and alcohol-attributable diseases and injuries; (3) incidence of alcohol-attributable diseases, injuries, and deaths; (4) population all-cause mortality; and (5) transition probabilities between the different drinking risk levels and the alcohol-attributable diseases, injuries and death. Participants’ daily consumption of alcohol and their self-reported quality of life at baseline, 12 weeks, 26 weeks, and 52 weeks post-baseline were sourced from the trial. General population’s all-cause mortality was sourced from Statistics Sweden [41]. The population all-cause mortality probabilities by age and sex can be seen in Table A7 in the appendix. Tables 5 and 6 summarize the utility and cost parameters and uncertainty ranges used in the model.

### Incidence of alcohol-attributable diseases, injuries, and deaths

Alcohol-related harmful events were identified and categorized based on pathophysiology and occurrence regarding alcohol consumption. These are listed and paired to the respective ICD-10 codes in Table A8 in the appendix.

Public data from registers held by the Swedish National Board of Health and Welfare were used to estimate the general population’s annual morbidity and mortality rates associated with the identified alcohol-related harmful events. Mortality was calculated using data from the *Cause of Death Register* [41]. Morbidity, expressed as the number of cases diagnosed with conditions resulting from alcohol consumption, was calculated using data from the patient register, including inpatient and specialized outpatient care [53]. ICD-10 codes were used to identify and cluster the episodes of care in both registers. Age-adjusted morbidity and mortality rates per 100,000 for 2020 were used. Mortality and morbidity rates obtained from this source can be found in Table A9 in the appendix.

### Utilities

Utility weights were assigned to each DRL state at the end of each cycle in the medium-term section of the model using the average utility value obtained from the trial subjects, contingent on gender, for each DRLs at the latest measurement time point where utilities were recorded (52 weeks). These utility values were derived from the EQ-5D-3 L questionnaire collected during the trial and were informed by the Swedish experience-based value sets for EQ-5D health states as reported by Burström et al. (2013) [54]. We encountered missing data due to participant attrition, with 15.0% missing data for BSCT and 16.67% for MET at 12 weeks, 30.0% missing data for BSCT and 27.78% for MET at 26 weeks, and 40.0% missing data for BSCT and 38.89% for MET at 52 weeks. Multiple imputation by chained equations (MICE) was used to handle the missing data, creating five imputed datasets. The imputation models included all relevant study variables to ensure accurate predictions. Diagnostic checks and sensitivity analyses confirmed the robustness and consistency of our results. Utility values for each alcohol-related event were obtained from a literature review, and these were adjusted to reflect the health effects of both the comorbidity and their underlying DRL condition [55]. Utility values can be seen in Tables 4 and 5.

**Table 4:** Utility weights for each drinking risk level during the trial

Drinking Risk Levels	Males	Females
**12 weeks**		
Low	0,850	0,834
Medium	0,840	0,857
High	0,842	0,872
Very High	NA	0,795
**26 weeks**		
Low	0,866	0,836
Medium	0,867	0,882
High	0,815	0,815
Very High	NA	0,718
**52 Weeks**		
Low	0,890	0,862
Medium	0,881	0,897
High	0,833	0,868
Very High	NA	NA

Note: NA = Not applicable due to there not being any individuals in the drinking risk level during the trial timepoint

**Table 5:** Utility values for alcohol-related events

	Utility		SE	Distribution Type	Source
Utilities					
**Medium Term (Non-reversible state)**					
Hemorrhagic stroke		0.45	0.03	Beta	Lanitis T et al., 2014 [56]
Cirrhosis of the liver		0.74	0.02	Beta	Asphaug L et al. 2020 [57]
Pancreatitis		0.34	0.07	Beta	Laramee P et al. 2013 [58]
Ischaemic heart disease		0.71	0.02	Beta	Korman M et al. 2017 [59]
Ischaemic stroke		0.65	0.31	Beta	Lindgren P et al. 2008 [60]
**Temporary State**					
Transport injuries		0.68	0.07	Beta	Franzen C et al., 2009 [61]
Injuries other than from transport		0.60	0.08	Beta	Wihlke G et al., 2021 [62]
Lower respiratory infections		0.75	0.20	Beta	Oppong R et al., 2016 [63]

### Cost analysis

#### Intervention costs

Intervention costs were obtained from trial cost logs. Initial training of eight psychologists, four nurses, two social workers, and three public health/behavioral scientists were seen as fixed costs for both intervention and comparator. Variable costs associated with the delivery of each treatment arm were also collected and computed. These included salaries of the health professionals, materials, rent of premises, coding for the measurement of adherence to MI principles, in the case of MET treatment, and overhead costs. (Table 6).

#### Cost offsets

The model estimated direct medical costs and indirect productivity losses for the alcohol-related events. To find the most recent data on these costs in a Swedish setting, or a context as similar as possible, a literature search of existing costing studies was performed in Advanced Google Scholar with the following terms: (Alcohol related event) AND (cost-effectiveness OR health-related quality of life OR health utility OR direct health costs OR indirect costs) AND (Sweden OR Denmark OR Norway OR Finland OR Germany OR Netherlands), conducted between January 2010 and July 2021. Extended searches going back until January 2000 or including other geographical regions were also performed if no appropriate data was identified in the initial search. All costs were converted to February 2024 United States dollar (USD) where needed using the CCEMG - EPPI-Centre Cost Converter v.1.4 [64]. The costs used in the analyses can be seen in Table 6. For several direct medical costs, a triangular distribution was employed in the probabilistic sensitivity analysis. The choice of a triangular distribution was guided by the nature of the available source data, which often provided estimates as a most likely value alongside a plausible range, rather than parameters like mean and standard deviation required for other distributions.

**Table 6:** Cost estimates per alcohol-related events (costs in 2024 USD)

	Mean	Uncertainty	Distribution Type^a^	Source
**Intervention Costs**				
MET	$1,511.37			Stockholm Centre for Dependency Disorders Trial
BSCT	$1,447.79			Stockholm Centre for Dependency Disorders Trial
**Costs of complications**				
**Direct costs**				
**MediumTerm (Non reversible state)**				
Haemorrhagic stroke	$26,139.98	$21,674.03 - $31,978.39	Triangular	Holm A et al., 2014 [65]
Cirrhosis of the liver	$14,416.73	$6,897.99 - $14,416.13	Triangular	Holm A et al., 2014 [65]
Pancreatitis	$10,080.83	$9,760.62 - $12,254.49	Triangular	Holm A et al., 2014 [65]
Ischemic heart disease	$9,536.14	$8,281.14 - $10,963.26	Triangular	Holm A et al., 2014 [65]
Ischemic stroke	$16,075.14	$15,450.44 - $16,383.98	Triangular	Holm A et al., 2014 [65]
Temporary State	$0.00	$149.22 - $2,107.18		
Transport injuries	$657.61	$154.55 - $3,261.29	Triangular	Holm A et al., 2014 [65]
Injuries other than from transport	$430.57	$154.55 - $3,261.29	Triangular	Holm A et al., 2014 [65]
Lower respiratory infections	$3,727.66			Wolf E et al. 2020 [66]
**Indirect costs (productivity losses)**				
**Medium Term (Nonreversible state)**				
Haemorrhagic stroke	$2,744.06		Gamma	Ghatnekar O et al., 2014 [67]
Cirrhosis of the liver	$15,328.40	$31,654.95 (SD)	Gamma	O’Hara J et al., 2020 [68]
Pancreatitis	$37,186.92	$50,244.56 (SD)	Gamma	Andersson, B et al. [69]
Ischemic heart disease	$15,624.96	$14,211.32 (SD)	Gamma	Zethraeus N et al., 2001 [70]
Ischemic stroke	$12,066.23	$8,301.84 - $16,088.81	Triangular	Lindgren P et al., 2008 [60]
**Temporary State**				
Transport injuries	$8,487.39			Papadakaki M et al., 2016 [71]
Injuries other than from transport	$7,047.08			Lindqvist K, Lindholm L, 2001 [72]
Lower respiratory infections	$4,233.10	$3,855.83 - $4,610.13	Gamma	Kirsch F et al., 2019 [73]

^a^ For triangular distributions, the ‘Mean’ value is the mode, and ‘Uncertainty’ provides the [min, max] range. This distribution is defined by these three parameters, with the probability density being zero at the min and max, and peaking at the mode

### Probabilistic sensitivity analyses

A probabilistic sensitivity analysis was conducted to account for statistical uncertainty in model parameters. Monte Carlo simulations with 1000 iterations were run to produce 95% uncertainty intervals around model outputs, using Visual Basic for Microsoft Excel 2019. Cost-effectiveness planes were used to depict the uncertainty surrounding the cost and QALY estimates for BSCT versus MET.

### Univariate sensitivity analyses

A set of univariate sensitivity analyses were performed to account for uncertainty in parameter assumptions used in model outputs. We modeled the following changes to base case assumptions: (a) assuming a relapse probability of 19% (14% in base case analysis) based on the estimates of Taylor and colleagues (relapse estimates from the study varied between 14% and 19%, where 19% pertained to individuals who aimed for abstinence); (b) assuming a discount rate of 0% and 5% respectively (3% in base case) to capture the potential variability in time preference rates and to assess the robustness of our results under different discounting scenarios; (c) removing the supervision costs from the intervention costs, (see base case costs in Table 8), and (d) using a General Anxiety Disorder (GAD) score converted to SF-6D utilities for alcohol risk drinking states [65].

## Results

### Base case cost-effectiveness results

The base case results can be seen in Table 7. The intervention costs for the MET and BSCT treatment arms were calculated at $1,511.12 and $1,448.21 per enrolled patient, respectively. BSCT incurred lower healthcare costs of alcohol-related complications than MET, with a difference of $-50.52 (95% UI: -$56.98 to -$43.30) on average per person. Although the 95% UIs for the total costs per arm in Table 7 may show overlap, this incremental UI, derived from paired calculations in the probabilistic sensitivity analysis, does not include zero, indicating that BSCT is consistently less costly than MET across the range of parameter uncertainty explored. Productivity costs associated with alcohol-related complications were also slightly lower in the BSCT arm compared to MET, with a difference of $-4.00 (95% UI: -$13.50 to $6.58) on average per person. Overall, BSCT led to lower total healthcare costs (incremental cost -$114.10; 95% UI: -$99.43 to -$118.22) and lower total societal costs (incremental cost -$118.12; 95% UI: -$166.23 to -$100.04) per person than MET. Furthermore, BSCT also resulted in more QALYs than MET, with a difference of 0.060 (95% UI: -0.05 to 0.07) on average per person. Table 7. Base case cost-effectiveness results comparing BSCT to MET, in 2024 USD per person.

**Table 7:** Base case cost-effectiveness results comparing BSCT to MET, in 2024 USD per person

	MET	BSCT	Incremental (BSCT vs. MET)
	Mean (95% UI)	Mean (95% UI)	Mean (95% UI)
Intervention Costs	$1,511.37	$1,447.79	-$63.60
Healthcare Costs	$2,585.56	$2,540.48	-$50.52
	($2,333.47 to $2,935.38)	($2,295.15 to $2,880.87)	(-56.98 to $-43.30)
Productivity Costs	$1,128.72	$1,124.71	-$4.00
	($926.01 to $1,363.16)	($915.15 to $1,366.83)	(-13.50 to $6.58)
Total Healthcare Costs	$4,100.16	$3,981.98	-$114.10
	($3,842.59 to $4,449.03)	($3,748.30 to $4,321.28)	(-99.43 to $-118.22)
Total Societal Costs	$5,224.37	$5,106.95	-$118.12
	($4,770.25 to $5,807.63)	($4,656.07 to $5,695.36)	(-166.23 to $-100.04)
QALYs	3.434	3.494	60
	(3.39 to 3.45)	(3.41 to 3.53)	(-0.05 to 0.07)
ICER (Societal Perspective)	Dominant		
ICER (Healthcare Perspective)	Dominant		

Note: UI – Uncertainty interval; ICER – Incremental Cost Effectiveness Ratio

Dominant – intervention yields more QALYs and lower costs than the comparator

*Intervention costs + healthcare costs of alcohol related complications

** Intervention costs + healthcare costs of alcohol related complications + productivity losses

**Fig. 2:**
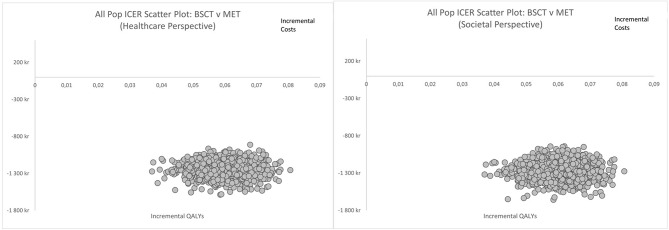
**a-b**. Cost effectiveness planes

The cost-effectiveness plane scatter plots (Figs. 2a-b) depict the uncertainty surrounding the cost and quality-adjusted life-year (QALY) estimates for BSCT versus MET. For both healthcare- and limited societal perspectives, all data points are consistently located in the southeast quadrant. This suggests that BSCT is less costly and more effective than MET from both the healthcare and limited societal perspectives.

### Univariate sensitivity analyses

Table 8 summarizes the results of the univariate sensitivity analyses. Results remained robust to most changes to base case assumptions. In the base case scenario, which accounts for 3% discounting (the process of reducing the value of future costs and benefits to reflect their present value), supervision costs, and a 14% decay rate, BSCT demonstrated better cost-effectiveness. Similar conclusions can be drawn when varying the discount rate; both at 0% and 5%, BSCT consistently showcased to be dominant compared with MET.

When coding costs were eliminated from the analysis, BSCT maintained similar cost advantages compared to MET. Incorporating a 19% decay rate also depicted BSCT as the dominant option, with costs and QALYs continuing to favor BSCT. The use of GAD-7 derived utility values again displayed BSCT’s dominance, reaffirming its apparent superior cost-per-QALY performance over MET.

**Table 8:** Univariate sensitivity analyses

			Incremental		Societal ICER	Healthcare ICER
**Base Case (3% Discounting. Supervision Costs Included. 14% decay rate)**	**Costs (USD)**	**QALYs**	**Costs**	**QALYs**	**(USD/QALY)**	**(USD/QALY)**
BSCT	$5,106.95	3,49	-$118.12	0,06	Dominant	Dominant
MET	$5,224.37	3,43				
**Discounting 0%**	**Costs (USD)**	**QALYs**	**Costs**	**QALYs**	**(USD/QALY)**	**(USD/QALY)**
BSCT	$5,532.97	3,81	-$126.42	0,07	Dominant	Dominant
MET	$5,659.39	3,74				
**Discounting 5%**	**Costs (USD)**	**QALYs**	**Costs**	**QALYs**	**(USD/QALY)**	**(USD/QALY)**
BSCT	$4,869.33	3,22	-$114.03	0,05	Dominant	Dominant
MET	$4,983.36	3,16				
**No Supervision Costs**	**Costs (USD)**	**QALYs**	**Costs**	**QALYs**	**(USD/QALY)**	**(USD/QALY)**
BSCT	$5,105.17	3,49	-$117.52	0,06	Dominant	Dominant
MET	$5,222.69	3,43				
**19% Decay Rate**	**Costs (USD)**	**QALYs**	**Costs**	**QALYs**	**(USD/QALY)**	**(USD/QALY)**
BSCT	$5,239.72	3,48	-$133.33	0,07	Dominant	Dominant
MET	$5,373.05	3,41				
**GAD 7 Derived Utility Values**	**Costs (USD)**	**QALYs**	**Costs**	**QALYs**	**(USD/QALY)**	**(USD/QALY)**
BSCT	$5,117.99	4,06	-$112.92	0,07	Dominant	Dominant
MET	$5,230.91	4,00				

Note: BSCT = Behavioral Self-Control Training; MET = Motivational Enhancement Therapy; GAD-7 = Generalized Anxiety Disorder Scale

## Discussion

AUD is a significant public health concern in Sweden, with a substantial portion of the population meeting criteria for the disorder. This study aimed to assess the cost-effectiveness of two well-established treatments for AUD; BSCT and MET, both with a treatment goal of controlled drinking.

The base case results demonstrated that BSCT was less expensive ($1,448.21) compared to MET ($1,511.12) per enrolled patient. BSCT participants also incurred lower healthcare and societal costs and marginally higher QALYs than MET. These findings are particularly noteworthy given that the underlying randomized controlled trial, from which the efficacy data were derived, found no statistically significant difference between BSCT and MET in their primary clinical outcome of mean weekly alcohol consumption at 26 weeks (t = -0.278, *P* = 0.781, d = -0.22, 95% CI = -0.60 to 0.16) [32]. Thus, in a context of comparable clinical effectiveness for the primary outcome, BSCT emerges as a dominant strategy, offering similar clinical benefits at a lower cost and with a slight QALY advantage. Sensitivity analyses under various scenarios, including changes in discount rates, coding costs, and decay rates, consistently favored BSCT, confirming its superior cost-effectiveness and cost-per-QALY performance over MET. This dominance means that BSCT is not only the preferred option but also that its probability of being cost-effective is 100% at any positive societal willingness-to-pay for health gains.

The results of our economic evaluation demonstrated several important findings. First, we observed that BSCT was associated with slightly lower intervention costs compared to MET, indicating direct cost savings per enrolled patient. This finding is a crucial factor in healthcare resource allocation, decision-making and priority setting [2].

Secondly, BSCT demonstrated further cost savings when compared to MET, from both a healthcare perspective, which considers direct medical costs, and a limited societal perspective, which includes associated costs such as lost productivity and informal care. This suggests that BSCT not only resulted in improved health outcomes, reflected in gained QALYs, but also reduced expenditures and productivity losses, compared to MET. Noteworthy is though, that despite the treatment manuals for BSCT and MET differed in pre-specified numbers of sessions (five for BSCT and four for MET), in practice, there were no significant differences between groups regarding number of sessions received at the 12 week follow-up. Participants in the BSCT group received an average of 3.8 sessions, and the MET group 3.6 sessions. This means that the estimations of cost-effectiveness infer on BSCT, being cost-effective when the interventions were equally delivered in both groups. This needs to be taken into consideration when considering future implementation and new guidelines of recommend treatments in AUD. This similarity in sessions attended, despite differing prescribed lengths, could be due to various factors including patient engagement, perceived need, or early dropout, and may have contributed to the comparable clinical effectiveness observed between the two interventions.

To our knowledge, there are no previous cost-effectiveness analyses (CEA) of BSCT. Several short-term CEAs of MET and other psychological AUD treatments have shown to improve both patient outcomes and yielded cost-savings within healthcare as well as other sectors [30, 31].

The UK Alcohol Treatment Trial (UKATT) revealed that social behavior and network therapy, similar to BSCT in its behavioral methodology, was equally effective as MET in enhancing patient outcomes, with no significant difference in overall costs [30]. Furthermore, an economic evaluation from a randomized controlled trial on MET with or without cognitive behavior therapy (CBT) for Type 1 diabetes suggests that these therapies, though not always cost-effective, could yield potential healthcare savings and enhancements in quality of life [66]. A review by J. Rehm et al. explored the cost-effectiveness of psychotherapeutic approaches, such as MET, in combination with pharmacological treatments in managing AUD [67]. They found that health economic research in this field is scarce, but that most treatment approaches are usually cost-effective, including those with behavioral components such as BSCT, offering a viable option for promoting moderated alcohol consumption among individuals with AUD [67]. Research comparing individual versus group female-specific CBT for AUD adds further insight, illustrating how the mode of treatment delivery (individual vs. group) can affect cost-effectiveness. This study found that individual sessions are likely to be cost-effective if the willingness to pay threshold is above 54 dollars for each fewer drinking day or heavy drinking day [68].

Another study exploring cost-effectiveness of treatments aimed at individuals with AUD suggests that, while combined pharmacological and psychologic treatments initially appear to be cost effective options from both healthcare sector and limited societal perspectives, probabilistic sensitivity analyses revealed the potential for other strategies, such as behavioral interventions only or combined with pharmacological treatment, to be cost-effective due to minor differences in remission and relapse rates. The limited societal perspective highlights broader benefits, including increased labor force participation and reduced crime rates, influencing cost-effectiveness [69]. Finally, a study exploring the cost-effectiveness of different alcohol use treatments among individuals with compensated alcohol-related cirrhosis demonstrates that short-term medication-assisted treatments and counseling, akin to behavioral interventions, are highly cost-effective and often result in cost savings when treating AUD across age groups, in comparison to no treatment [70]. 

Our results suggest that BSCT could be a cost-effective alternative for achieving controlled drinking in AUD. This finding is particularly significant given the ongoing debate regarding the optimal treatment goals for AUD, whether abstinence or controlled drinking [10, 71]. Our findings, therefore, may be used in continuous discussions and as input for future updates of treatment guidelines.

### Strengths and limitations

Our study has several strengths, including the use of a model that accounts for the potential medium-term complications related to AUD, and the related costs and quality of life impacts. Additionally, the trial population characteristics are similar to the characteristics of the majority of individuals with AUD, that is, moderately dependent and with low levels of psychiatric comorbidities [2].

This study has several limitations worth noting. The 5-year time horizon considered in the model may not account for the longer-term consequences of AUD or the potential relapses after this period. Regarding the study population, the high proportion of employed individuals, coupled with baseline utility values that appear relatively high and potentially approach age-matched population norms, suggests our sample was relatively high-functioning. While these characteristics might limit generalizability to individuals with more severe AUD and associated functional impairment, they are consistent with a treatment-seeking group aiming for controlled drinking. Consequently, our utility values were subject to uncertainty due to the low sample size for the high and very high-risk individuals at the different timepoints. This had the results that individuals at higher risk levels reported higher utility values than those at lower risk levels.

The relatively high baseline utilities could also indicate limitations in the sensitivity of the EQ-5D-3 L in this specific AUD population, although the cost-effectiveness findings are driven by the incremental differences between BSCT and MET. Furthermore, the data on the effectiveness of MET and BSCT was derived from a single randomized controlled trial (RCT) rather than from meta-analyses. Using data from a single trial may limit the generalizability of the findings, as meta-analyses typically provide more robust evidence by pooling results from multiple studies.

To further enhance the relevance of our findings, future research should consider comparing the results of BSCT and MET with cohorts receiving no treatment or pursuing complete abstinence-based treatments. This comparison would provide a more comprehensive understanding of the relative effectiveness and cost-effectiveness of these interventions in the context of AUD management. Matching participants’ goals with treatment objectives remains a vital factor in evaluating the efficacy of AUD treatments, and future studies should continue to explore this aspect to tailor interventions effectively.

Additionally, exploring the possibility of extending the time horizon, with careful consideration of assumptions, could shed light on the long-term effects and cost-effectiveness of these treatments. Future research could explore the potential cost-effectiveness of targeting different treatment goals, for instance, abstinence versus controlled drinking.

## Conclusions

This study assessed the cost-effectiveness of BSCT and MET for individuals with AUD aiming for controlled drinking. Our findings indicate that BSCT is associated with cost savings and improved health outcomes compared to MET. These results highlight the economic advantages of BSCT, suggesting that it not only yields positive health outcomes but also reduces healthcare expenditures and limited societal costs.

From a clinical perspective, providing cost-effective treatments like BSCT can result in optimally utilized resources, potentially allowing more individuals to receive care within the same budget. Given that BSCT and MET result in similar outcomes in reducing alcohol consumption and related consequences [32], the cost-effectiveness of BSCT offers valuable guidance for treatment implementation decisions.

## Electronic supplementary material

Below is the link to the electronic supplementary material.

Supplementary Material 1

## Data Availability

Input used in the model are available from the corresponding author on reasonable request.
