# Supplementary material for: A cost-effectiveness analysis of two psychological treatments for controlled drinking in individuals with alcohol use disorder
Source: Cost Eff Resour Alloc. 2025 Jun 2;23:24. doi: 10.1186/s12962-025-00633-9 (PMC12131623; doi:10.1186/s12962-025-00633-9)
Supplement: Supplementary file 1 — Supplementary Material 1 [file 12962_2025_633_MOESM1_ESM.docx]

APPENDIX

Table A1. Annual Morbidity Probability for Alcohol Related Events according to WHO Drinking Risk Levels.

| *Morbidity: Yearly transitional probabilities to postevent state (Year 2+):* | | | | | |
| --- | --- | --- | --- | --- | --- |
| Post-event states |  |  |  |  |  |
|  | Hemorrhagic Stroke | Liver Chirrosis | Acute Pancreatis | Ischemic Heart Disease | Ischemic Stroke |
| Men Low Risk | 0.094% | 0.085% | 0.101% | 0.612% | 0.242% |
| Men Medium Risk | 0.108% | 0.154% | 0.145% | 0.520% | 0.279% |
| Men High Risk | 0.127% | 0.297% | 0.219% | 0.819% | 0.325% |
| Men Very High Risk | 0.170% | 0.964% | 0.454% | 0.993% | 0.422% |
| Men (Normal Population) | 0.077% | 0.038% | 0.061% | 0.819% | 0.252% |
|  |  |  |  |  |  |
|  |  |  |  |  |  |
|  | Hemorrhagic Stroke | Liver Chirrosis | Chronic Pancreatitis | Ischemic Heart Disease | Ischemic Stroke |
| Women Low Risk | 0.090% | 0.283% | 0.040% | 0.346% | 0.155% |
| Women Medium Risk | 0.125% | 0.514% | 0.078% | 0.435% | 0.228% |
| Women High Risk | 0.198% | 0.973% | 0.215% | 0.582% | 0.459% |
| Women Very High Risk | 0.310% | 1.618% | 0.523% | 0.773% | 0.990% |
| Women (Normal Population) | 0.061% | 0.053% | 0.051% | 0.366% | 0.211% |
| Tunnel States |  |  |  |  |  |
|  | Lower Respiratory Infection | Transport Injuries | Injuries other than from transport |  |  |
| Men Low Risk | 0.781% | 0.132% | 2.739% |  |  |
| Men Medium Risk | 0.863% | 0.168% | 3.650% |  |  |
| Men High Risk | 0.966% | 0.207% | 4.456% |  |  |
| Men Very High Risk | 1.181% | 0.267% | 5.400% |  |  |
| Men (Normal Population) | 0.679% | 0.054% | 0.877% |  |  |
|  |  |  |  |  |  |
| Women Low Risk | 0.733% | 0.113% | 2.361% |  |  |
| Women Medium Risk | 0.815% | 0.150% | 3.290% |  |  |
| Women High Risk | 0.947% | 0.197% | 4.265% |  |  |
| Women Very High Risk | 1.095% | 0.239% | 4.903% |  |  |
| Women (Normal Population) | 0.645% | 0.048% | 0.775% |  |  |

Table A2. Three Month Morbidity Probability for Alcohol Related Events according to WHO Drinking Risk Levels.

| *Morbidity: Three-month transitional probabilities to postevent state (Year 1)* | | | | | |
| --- | --- | --- | --- | --- | --- |
| Post-event states |  |  |  |  |  |
|  |  | *Formula for three-month probabilities = 1-(1-Yearly Probability) ^(months/year)* | | | |
|  | Hemorrhagic Stroke | Liver Chirrosis | Chronic Pancreatis | Ischemic Heart Disease | Ischemic Stroke |
| Men Low Risk | 0.023% | 0.021% | 0.025% | 0.153% | 0.061% |
| Men Medium Risk | 0.027% | 0.038% | 0.036% | 0.130% | 0.070% |
| Men High Risk | 0.032% | 0.074% | 0.055% | 0.205% | 0.081% |
| Men Very High Risk | 0.043% | 0.242% | 0.114% | 0.249% | 0.106% |
| Men (Normal Population) | 0.019% | 0.009% | 0.015% | 0.205% | 0.063% |
|  |  |  |  |  |  |
|  | Hemorrhagic Stroke | Liver Chirrosis | Chronic Pancreatis | Ischemic Heart Disease | Ischemic Stroke |
| Women Low Risk | 0.023% | 0.071% | 0.010% | 0.087% | 0.039% |
| Women Medium Risk | 0.031% | 0.129% | 0.020% | 0.109% | 0.057% |
| Women High Risk | 0.050% | 0.244% | 0.054% | 0.146% | 0.115% |
| Women Very High Risk | 0.078% | 0.407% | 0.131% | 0.194% | 0.248% |
| Women (Normal Population) | 0.015% | 0.013% | 0.013% | 0.092% | 0.053% |
| Tunnel States |  |  |  |  |  |
|  | Lower Respiratory Infection | Transport Injuries | Injuries other than from transport |  |  |
| Men Low Risk | 0.196% | 0.033% | 0.692% |  |  |
| Men Medium Risk | 0.217% | 0.042% | 0.925% |  |  |
| Men High Risk | 0.242% | 0.052% | 1.133% |  |  |
| Men Very High Risk | 0.297% | 0.067% | 1.378% |  |  |
| Men (Normal Population) | 0.170% | 0.014% | 0.220% |  |  |
|  |  |  |  |  |  |
| Women Low Risk | 0.184% | 0.028% | 0.595% |  |  |
| Women Medium Risk | 0.204% | 0.038% | 0.833% |  |  |
| Women High Risk | 0.238% | 0.049% | 1.084% |  |  |
| Women Very High Risk | 0.275% | 0.060% | 1.249% |  |  |
| Women (Normal Population) | 0.162% | 0.012% | 0.194% |  |  |

Table A3. Annual Mortality Probability for Alcohol Related Events according to WHO Drinking Risk Levels.

| *Mortality: Yearly transitional probabilities to postevent state (Year 2+):* | | | | | | | | | |
| --- | --- | --- | --- | --- | --- | --- | --- | --- | --- |
| Postevent states |  | |  | |  | |  | |  |
|  | Hemorrhagic Stroke | | Liver Chirrosis | | Chronic Pancreatis | | Ischemic Heart Disease | | Ischemic Stroke |
| Men Low Risk | 0.015% | | 0.007% | | 0.002% | | 0.086% | | 0.021% |
| Men Medium Risk | 0.018% | | 0.012% | | 0.003% | | 0.073% | | 0.025% |
| Men High Risk | 0.024% | | 0.043% | | 0.007% | | 0.115% | | 0.033% |
| Men Very High Risk | 0.024% | | 0.043% | | 0.007% | | 0.115% | | 0.033% |
| Men (Normal Population) | 0.013% | | 0.003% | | 0.001% | | 0.115% | | 0.022% |
|  |  | |  | |  | |  | |  |
|  |  | |  | |  | |  | |  |
|  | Hemorrhagic Stroke | | Liver Chirrosis | | Chronic Pancreatis | | Ischemic Heart Disease | | Ischemic Stroke |
| Women Low Risk | 0.014% | | 0.011% | | 0.001% | | 0.052% | | 0.013% |
| Women Medium Risk | 0.020% | | 0.020% | | 0.001% | | 0.066% | | 0.019% |
| Women High Risk | 0.040% | | 0.050% | | 0.007% | | 0.101% | | 0.056% |
| Women Very High Risk | 0.040% | | 0.050% | | 0.007% | | 0.101% | | 0.056% |
| Women (Normal Population) | 0.010% | | 0.002% | | 0.001% | | 0.055% | | 0.018% |
| *Yearly transitional probabilities to tunnel state (Year 2+)* | | | | | | | |  |  |
| Tunnel states | |  | |  | |  | |  |  |
|  | | Lower Respiratory Infection | | Transport Injuries | | Injuries other than from transport | |  |  |
| Men Low Risk | | 0.021% | | 0.039% | | 0.003% | |  |  |
| Men Medium Risk | | 0.023% | | 0.055% | | 0.005% | |  |  |
| Men High Risk | | 0.028% | | 0.087% | | 0.007% | |  |  |
| Men Very High Risk | | 0.028% | | 0.087% | | 0.007% | |  |  |
| Men (Normal Population) | | 0.018% | | 0.054% | | 0.877% | |  |  |
|  | |  | |  | |  | |  |  |
|  | |  | |  | |  | |  |  |
|  | | Lower Respiratory Infection | | Transport Injuries | | Injuries other than from transport | |  |  |
| Women Low Risk | | 0.011% | | 0.018% | | 0.001% | |  |  |
| Women Medium Risk | | 0.012% | | 0.026% | | 0.001% | |  |  |
| Women Hi ghRisk | | 0.015% | | 0.042% | | 0.002% | |  |  |
| Women Very High Risk | | 0.015% | | 0.042% | | 0.002% | |  |  |
| Women (Normal Population) | | 0.010% | | 0.007% | | 0.000% | |  |  |

Table A4. Three Month Mortality Probability for Alcohol Related Events according to WHO Drinking Risk Levels.

| *Mortality: Three Month transitional probabilities to postevent state (Year 1):* | | | | | |
| --- | --- | --- | --- | --- | --- |
| Postevent states |  |  | *Formula for three-month probabilities = 1-(1-Yearly Probability) ^(months/year)* | | |
|  | Hemorrhagic Stroke | Liver Chirrosis | Chronic Pancreatis | Ischemic Heart Disease | Ischemic Stroke |
| Men Low Risk | 0.004% | 0.002% | 0.001% | 0.022% | 0.005% |
| Men Medium Risk | 0.004% | 0.003% | 0.001% | 0.018% | 0.006% |
| Men High Risk | 0.005% | 0.006% | 0.001% | 0.029% | 0.007% |
| Men Very High Risk | 0.007% | 0.019% | 0.002% | 0.035% | 0.009% |
| Men (Normal Population) | 0.003% | 0.001% | 0.000% | 0.029% | 0.006% |
|  |  |  |  |  |  |
|  |  |  |  |  |  |
|  | Hemorrhagic Stroke | Liver Chirrosis | Chronic Pancreatis | Ischemic Heart Disease | Ischemic Stroke |
| Women Low Risk | 0.004% | 0.003% | 0.000% | 0.013% | 0.003% |
| Women Medium Risk | 0.005% | 0.005% | 0.000% | 0.016% | 0.005% |
| Women High Risk | 0.010% | 0.013% | 0.002% | 0.025% | 0.014% |
| Women Very High Risk | 0.010% | 0.013% | 0.002% | 0.025% | 0.014% |
| Women (Normal Population) | 0.002% | 0.001% | 0.000% | 0.014% | 0.004% |
| *Three-month transitional probabilities to tunnel state (Year 1)* | | | |  | |
| Tunnel states |  |  |  |  | |
|  |  | *Formula for three-month probabilities = 1-(1-Yearly Probability) ^(months/year)* | | | |
|  | Lower Respiratory Infection | Transport Injuries | Injuries other than from transport |  | |
| Men Low Risk | 0.005% | 0.010% | 0.001% |  | |
| Men Medium Risk | 0.006% | 0.014% | 0.001% |  | |
| Men High Risk | 0.007% | 0.022% | 0.002% |  | |
| Men Very High Risk | 0.007% | 0.022% | 0.002% |  | |
| Men (Normal Population) | 0.004% | 0.014% | 0.220% |  | |
|  |  |  |  |  | |
|  |  |  |  |  | |
|  | Lower Respiratory Infection | Transport Injuries | Injuries other than from transport |  | |
| Women Low Risk | 0.003% | 0.004% | 0.000% |  | |
| Women Medium Risk | 0.003% | 0.006% | 0.000% |  | |
| Women High Risk | 0.004% | 0.010% | 0.000% |  | |
| Women Very High Risk | 0.004% | 0.010% | 0.000% |  | |
| Women (Normal Population) | 0.002% | 0.002% | 0.000% |  | |

**Table A5.** Full list of Trial Inclusion and Exclusion Criteria

| Inclusion Criteria: |
| --- |
| Patients who meet the criteria for alcohol use syndrome according to DSM 5. |
| Between 18 and 70 years of age |
| Stated goal that reduce current alcohol consumption |
| Alcohol consumption at least 30 of the last 90 the days before the screening |
| Living in Stockholm or Västra Götaland |
| Willingness to give informed consent. |
| Exclusion Criteria |
| Sobriety as a goal of treatment |
| Severe somatic or psychiatric problems |
| Pregnancy |
| Current use of drugs |
| Harmful use or dependence on a substance other than alcohol (except nicotine) |
| Cared for the last 12 months for alcohol intoxication |
| Alcohol withdrawal or other serious alcohol-related harm |
| Elevated values of any of the liver samples AST. ALT. GT |
| Other treatment for alcohol problems in the last 3 months |
| Clearly indicated interest in pharmacological treatment for alcohol problems. |

**Table A6.** Baseline Characteristics of Population

| Gender | Man | 132 |
| --- | --- | --- |
|  | Woman | 120 |
| Civil Status | Single | 46 |
|  | Sambo* | 62 |
|  | Sarbo** | 22 |
|  | Married | 100 |
|  | Separated | 17 |
|  | Widowed | 2 |
| Have Kids | Yes | 193 |
|  | No | 56 |
| Housing form | Independent living | 237 |
|  | Sublease / Resident | 12 |
| Accommodation type | Tenancy | 67 |
|  | Condominium | 107 |
|  | Villa or townhouse | 74 |
|  | Other accommodation | 1 |
| Education | Incomplete primary school | 1 |
|  | Elementary school | 8 |
|  | Upper secondary school | 41 |
|  | Post-secondary studies | 26 |
|  | Studies at university / college less than 3 years | 47 |
|  | Studies at university / college 3 years or more | 125 |
| Employment | Employee | 145 |
|  | Self-employed | 61 |
|  | Leave of absence or parental leave | 1 |
|  | Studying. practicing | 2 |
|  | Labor market measures | 0 |
|  | Unemployed | 5 |
|  | Retirement pension | 26 |
|  | Sickness / activity compensation | 7 |
|  | Long-term sick leave | 0 |
|  | Home worker. takes care of the household | 1 |
|  | Other employment | 0 |

* **Sambo**: Cohabiting partner

****Sarbo:** Partner living separately

**Table A7.** All-Cause Mortality Rates Sweden 2020

| All-Cause Mortality Rates. Sweden 2020. 20-90 Years Old | | | | | |
| --- | --- | --- | --- | --- | --- |
| Age | **Men** | **Women** | **Age** | **Men** | **Women** |
| 20 | 0.0007 | 0.0024 | **55** | 0.003565 | 0.002079 |
| 21 | 0.000523 | 0.000209 | **56** | 0.004207 | 0.002206 |
| 22 | 0.000507 | 0.000112 | **57** | 0.004287 | 0.00226 |
| 23 | 0.000591 | 0.000292 | **58** | 0.004961 | 0.003398 |
| 24 | 0.000679 | 0.000242 | **59** | 0.005671 | 0.003598 |
| 25 | 0.000778 | 0.000241 | **60** | 0.006213 | 0.003526 |
| 26 | 0.000647 | 0.000281 | **61** | 0.006474 | 0.004561 |
| 27 | 0.00058 | 0.000242 | **62** | 0.007548 | 0.004358 |
| 28 | 0.000824 | 0.000232 | **63** | 0.008152 | 0.005073 |
| 29 | 0.000603 | 0.000147 | **64** | 0.009664 | 0.006112 |
| 30 | 0.000683 | 0.000299 | **65** | 0.010818 | 0.00635 |
| 31 | 0.000517 | 0.000475 | **66** | 0.011425 | 0.007412 |
| 32 | 0.000588 | 0.000317 | **67** | 0.012532 | 0.008812 |
| 33 | 0.000718 | 0.000258 | **68** | 0.014428 | 0.008359 |
| 34 | 0.00068 | 0.000395 | **69** | 0.016252 | 0.009626 |
| 35 | 0.000591 | 0.000344 | **70** | 0.017602 | 0.011413 |
| 36 | 0.000671 | 0.000496 | **71** | 0.019218 | 0.0121 |
| 37 | 0.00075 | 0.00038 | **72** | 0.02038 | 0.014543 |
| 38 | 0.000597 | 0.000649 | **73** | 0.02251 | 0.016107 |
| 39 | 0.000659 | 0.000591 | **74** | 0.026373 | 0.016895 |
| 40 | 0.001024 | 0.00048 | **75** | 0.028622 | 0.019531 |
| 41 | 0.000797 | 0.000528 | **76** | 0.031652 | 0.021555 |
| 42 | 0.001031 | 0.000709 | **77** | 0.035083 | 0.024046 |
| 43 | 0.000973 | 0.0008 | **78** | 0.038611 | 0.026637 |
| 44 | 0.000934 | 0.000844 | **79** | 0.046249 | 0.031316 |
| 45 | 0.001316 | 0.000875 | **80** | 0.054193 | 0.036698 |
| 46 | 0.001337 | 0.000975 | **81** | 0.058604 | 0.043057 |
| 47 | 0.001574 | 0.000962 | **82** | 0.072499 | 0.047101 |
| 48 | 0.001478 | 0.000767 | **83** | 0.077709 | 0.055018 |
| 49 | 0.001636 | 0.00139 | **84** | 0.089663 | 0.063691 |
| 50 | 0.002092 | 0.001349 | **85** | 0.105417 | 0.07349 |
| 51 | 0.002174 | 0.001522 | **86** | 0.12148 | 0.081657 |
| 52 | 0.002634 | 0.001601 | **87** | 0.140183 | 0.096773 |
| 53 | 0.002815 | 0.001763 | **88** | 0.158075 | 0.118369 |
| 54 | 0.003211 | 0.001947 | **89** | 0.176959 | 0.136813 |
|  |  |  | **90** | 0.2102 | 0.144954 |
|  |  |  |  |  |  |

Table A8. Alcohol-attributable events with their respective ICD 10 Codes.

|  | **ICD-10 Code** |
| --- | --- |
| **Serious** | |
| Ischemic Heart Disease | I20-I25 |
| Ischemic Stroke | I63-I66 |
| Haemorrhagic Stroke | I60-I62 |
| Cirrhosis | K70. K74. K71.1 |
| Pancreatitis | K85 |
| **Temporary** | |
| Lower Respiratory Infections | J10-J18. J20-J22 |
| Transport Injuries | V021-V029. V031-V039. V041-V049. V092. V093. V123-V129. V133-V139. V143-V149. V194-V196. V203-V209. V213- V219. V223-V229. V233-V239. V243-V249.V253-V259. V263-V269. V273- V279. V283-V289. V294-V299. V304-V309.  V314-V319. V324-V329. V334-V339. V344-V349. V354-V359. V364-V369. V374-V379. V384-V389. V394-V399. V404-V409.  V414-V419. V424-V429. V434-V439. V444-V449. V454-V459. V464- V469. V474-V479. V484-V489. V494-V499. V504- V509. V514-V519. V524-V529. V534-V539. V544-V549. V554-V559. V564-V569. V574-V579. V584-V589. V594-V599.  V604-V609. V614-V619. V624-V629. V634-V639. V644-V649. V654- V659. V664-V669. V674-V679. V684-V689. V694- V699. V704-V709. V714-V719. V724-V729. V734-V739. V744-V749. V754-V759. V764-V769. V774-V779. V784-V789.  V794-V799. V803-V805. V811. V821. V830-V833. V840-V843. V850-V853. V860-V863. V870-V878. V892. |
| Injuries other than from transport | X40-X49. W00-W19. X00-X09. W65-W74. rest of V-series. and W20-W64. W 75-W99. X10-X39. X50-X59. Y40-Y86. Y88.  and Y89 (rest of V = V-series minus those listed under Transport Injuries. X60-X84 and Y87.0. X85-Y09. Y87.1 |

**TABLE A9.** Alcohol-related general population morbidity and mortality risks, 2020

|  | Males | Females |
| --- | --- | --- |
| Morbidity | | |
| *Serious* |  |  |
| Ischemic heart disease | 0.006793 | 0.006453 |
| Ischemic stroke | 0.002522 | 0.002107 |
| Hemorrhagic stroke | 0.000765 | 0.00061 |
| Cirrhosis of the liver | 0.000375 | 0.000525 |
| Acute Pancreatitis | 0.000607 | 0.00051 |
| *Temporary* |  |  |
| Lower respiratory infections | 0.006793 | 0.006453 |
| Transport injuries | 0.004343 | 0.003833 |
| Injuries other than from transport | 0.070186 | 0.061976 |
|  |  |  |
| Mortality | | |
| *Serious* |  |  |
| Ischemic heart disease | 0.0011518 | 0.0005509 |
| Ischemic stroke | 0.0002234 | 0.0001758 |
| Hemorrhagic stroke | 0.0001265 | 0.0000971 |
| Cirrhosis of the liver | 0.0000298 | 0.0000209 |
| Acute Pancreatitis | 0.0000132 | 0.0000097 |
| *Temporary* |  |  |
| Lower respiratory infections | 0.0001786 | 0.0000972 |
| Transport injuries | 0.0011327 | 0.0005365 |
| Injuries other than from transport | 0.0000635 | 0.0000166 |

## Table A10. Alcohol-related harmful events costs

| Event | Author | Year | Country | Setting | Cost estimate | Uncertainty | Components | Source | Currency | Year | Link | Note |
| --- | --- | --- | --- | --- | --- | --- | --- | --- | --- | --- | --- | --- |
| *Serious (Non-reversible state)* | | |  |  |  |  |  |  |  |  |  |  |
| Hemorrhagic stroke | Holm A. L.. Veerman L. Cobiac L. et al | 2014 | Denmark | Inpatient. outpatient | € 24 525.00 | 20335 - 30003 | Treatment. healthcare personnel. medication | Cost-Effectiveness of Preventive Interventions to Reduce Alcohol Consumption in Denmark | Euros | 2014 | [Link](https://journals.plos.org/plosone/article?id=10.1371/journal.pone.0088041) | Costs vary within sex and age brackets. I used men under 50 years as the cost estimate and included the least and most expensive values from different age and gender values for the disease as upper and lower limits of the uncertainty |
| Cirrhosis of the liver | Holm A. L.. Veerman L. Cobiac L. et al | 2014 | Denmark | Inpatient. outpatient | € 13 526.00 | 6472 - 13526 | Treatment. healthcare personnel. medication | Cost-Effectiveness of Preventive Interventions to Reduce Alcohol Consumption in Denmark | Euros | 2014 | [Link](https://journals.plos.org/plosone/article?id=10.1371/journal.pone.0088041) | Costs vary within sex and age brackets. I used men under 50 years as the cost estimate and included the least and most expensive values from different age and gender values for the disease as upper and lower limits of the uncertainty |
| Pancreatitis | Holm A. L.. Veerman L. Cobiac L. et al | 2014 | Denmark | Inpatient. outpatient | € 9 458.00 | 9158- 11498 | Treatment. healthcare personnel. medication | Cost-Effectiveness of Preventive Interventions to Reduce Alcohol Consumption in Denmark | Euros | 2014 | [Link](https://journals.plos.org/plosone/article?id=10.1371/journal.pone.0088041) | Costs vary within sex and age brackets. I used men under 50 years as the cost estimate and included the least and most expensive values from different age and gender values for the disease as upper and lower limits of the uncertainty |
| Ischaemic heart disease | Holm A. L.. Veerman L. Cobiac L. et al | 2014 | Denmark | Inpatient. outpatient | € 8 947.00 | 7770-10286 | Treatment. healthcare personnel. medication | Cost-Effectiveness of Preventive Interventions to Reduce Alcohol Consumption in Denmark | Euros | 2014 | [Link](https://journals.plos.org/plosone/article?id=10.1371/journal.pone.0088041) | Costs vary within sex and age brackets. I used men under 50 years as the cost estimate and included the least and most expensive values from different age and gender values for the disease as upper and lower limits of the uncertainty |
| Ischaemic stroke | Holm A. L.. Veerman L. Cobiac L. et al | 2014 | Denmark | Inpatient. outpatient | € 15 082.00 | 14496-15372 | Treatment. healthcare personnel. medication | Cost-Effectiveness of Preventive Interventions to Reduce Alcohol Consumption in Denmark | Euros | 2014 | [Link](https://journals.plos.org/plosone/article?id=10.1371/journal.pone.0088041) | Costs vary within sex and age brackets. I used men under 50 years as the cost estimate and included the least and most expensive values from different age and gender values for the disease as upper and lower limits of the uncertainty |
| *Temporary State* | |  |  |  |  |  |  |  |  |  |  |  |
| Transport injuries | Holm A. L.. Veerman L. Cobiac L. et al | 2014 | Denmark | Inpatient. outpatient | € 617.00 | 140-1977 | Treatment. healthcare personnel. medication | Cost-Effectiveness of Preventive Interventions to Reduce Alcohol Consumption in Denmark | Euros | 2014 | [Link](https://journals.plos.org/plosone/article?id=10.1371/journal.pone.0088041) | Costs vary within sex and age brackets. I used men under 50 years as the cost estimate and included the least and most expensive values from different age and gender values for the disease as upper and lower limits of the uncertainty |
| Injuries other than from transport | Holm A. L.. Veerman L. Cobiac L. et al | 2014 | Denmark | Inpatient. outpatient | € 404.00 | 145- 3060 | Treatment. healthcare personnel. medication | Cost-Effectiveness of Preventive Interventions to Reduce Alcohol Consumption in Denmark | Euros | 2014 | [Link](https://journals.plos.org/plosone/article?id=10.1371/journal.pone.0088041) | Cost used was men from 50-69 years. as the value for men under 50 was negative due to regression techniques used to estimate costs |
| Lower respiratory infections | Wolff E. Storsaeter J. Ortqvist A | 2020 | Sweden (Jonkoping) | In and outpatient | € 3 497.40 | None given | Treatment. healthcare personnel. medication | Cost-effectiveness of pneumococcal vaccination for elderly in Sweden | Euros | 2020 | [Link](https://www.sciencedirect.com/science/article/pii/S0264410X20307271) | Includes data for both in and outpatient. Addition was done manually. The costs were done on 65 and 75-year-old patients |
| Lower respiratory infections | Oppong R. Coast J. Hood K et al | 2010 | Sweden (Jonkoping) | Outpatient | € 116.47 | (34.29 SD) | Staff. Hospitalization. Testing with X ray and Blood Work | Resource use and costs of treating acute cough/lower respiratory tract infections in 13 European countries: results and challenges | Euros | 2010 | [Link](https://link.springer.com/article/10.1007/s10198-010-0239-1) | Only outpatient cost data in primary care. which makes these costs extremely low compared to our guiding study. Other studies show similar values. and I am having a hard time finding hospitalized adults LRI cost data. |

## Table A11. Alcohol-Related Harmful Events Utilities

| Event | | | Sample Composition | | | | | Source | | | | Note |  |  |
| --- | --- | --- | --- | --- | --- | --- | --- | --- | --- | --- | --- | --- | --- | --- |
|  | **Utility** | **Uncertainty** | **Sample Size** | **Country** | **Age** | **Female** | **Male** | **Authors** | **Year** | **Link** | |  |  |  |
| *Serious (Non-reversible state)* | | | | | | | | | | | | |  |  |
| Hemorrhagic stroke | **0.4445** | (0.387 – 0.504) | 23799 (Two trials. ARISTOTLE and AVERROES) | Over 40 countries | Mean: 70 | ≈40% | ≈60% | Lanitis T. Kongnakorn T | 2014 | [Link](https://www.sciencedirect.com/science/article/pii/S004938481400293X#bb0170) | Sweden-specific study used this data for cost-effectiveness analysis | |  |  |
| Cirrhosis of the liver (Compensated) | **0.88** | 0.85 - 0.91 | 37 | USA | Mean:52 | 67% | 47% | Asphaug L. Thiele M. Krag A | 2004 | [Link](https://aasldpubs.onlinelibrary.wiley.com/doi/full/10.1002/hep.30979) | | Sweden-specific study used this data for cost effectiveness analysis |  |  |
| Cirrhosis of the liver (Decompensated. not severe) | **0.74** | 0.70-0.78 | 59 | USA | Mean:53 | 67% | 47% | Asphaug L. Thiele M. Krag A | 2004 | [Link](https://aasldpubs.onlinelibrary.wiley.com/doi/full/10.1002/hep.30979) | | Sweden-specific study used this data for cost effectiveness analysis |  |  |
| Cirrhosis of the liver (Decompensated. severe) | **0.55** | 0.51-0.59 | 18 | USA | Mean:54 | 67% | 47% | Asphaug L. Thiele M. Krag A | 2004 | [Link](https://aasldpubs.onlinelibrary.wiley.com/doi/full/10.1002/hep.30979) | | Sweden-specific study used this data for cost effectiveness analysis |  |  |
| Pancreatitis (Chronic with distal obstruction of pancreatic duct chosen due to continuous drinking) | **0.335** | SEM=0.069 | 39 | Netherlands | ≈ Mean 50 | ≈66% | ≈33% | Laramee P. Wonderling D. Cahen D et al | 2013 | [Link](https://bmjopen.bmj.com/content/3/9/e003676) | | Netherlands. surgery arm utility at baseline was used because it had alcohol users) Seems low because it is an acute episode of a chronic condition |  |  |
| Ischaemic heart disease (acute. event-11 days) | **0.71** | (0.08) SE | Traced back to a Norwegian doctoral dissertation 2012. no access is possible | | | | | Korman M. Wisloff T | 2017 | [Link](https://academic.oup.com/ehjcvp/article/4/1/15/3746062?login=true) | | Norway Data | |  |
| Post Ischemic Heart Disease (chronic) | **0.8** | (0.20) SE | 754 | Norway | ≈Mean 64 | ≈30% | ≈70% | Korman M. Wisloff T | 2017 | [Link](https://academic.oup.com/ehjcvp/article/4/1/15/3746062?login=true) | | Norway Data | |  |
| Ischaemic stroke | **0.65** | (0.31) SD | 57 | Sweden | Mean 63.2 | 44.30% | 55.70% | Lindgren P. Glader E. Jönson B | 2008 | [Link](https://academic.oup.com/eurjpc/article/15/2/230/5933055?login=true) | | Swedish specific utility score within 3 months of event |  |  |
| *Temporary* | | | | | | | | | | | | |  |  |
| Transport injuries | **0.68** | (0.32) SD | 81 | Sweden | Mean: 38.63 | 57% | 43% | Franzen C. Björnstig. Brulin C | 2009 | [Link](https://link.springer.com/article/10.1186/1472-6963-9-98/tables/1) | | Swedish specific. assuming car occupancy. For bikes and people walking it is worse |  |  |
| Injuries other than from transport | **0.6** | (0.35) SD | 123 | Sweden | 18-64 | ≈25% | ≈75% | Wihlke G. Strömmer L. | 2021 | [Link](https://link.springer.com/article/10.1007/s00068-019-01170-w/tables/3) | | Swedish-specific value after 3 months of traumatic injury |  |  |
| Lower respiratory infections | **0.752** | 0.192 SD | 2060 | 12 EU countries: Belgium. France. Germany. Italy. Netherlands. Poland. Slovakia. Slovenia. Spain. Sweden and the UK (England and Wales) | Mean: 49 | 59.41% | 40.60% | Oppong R. Smith R. Little p | 2016 | [Link](https://bjgp.org/content/bjgp/66/650/e633.full.pdf) | | Patients from across 12 European countries. including Sweden. only outpatient data |  |  |

Table A12. Alcohol-related general population risks, 2020

|  | | |
| --- | --- | --- |
|  | **Males** | **Females** |
| **Morbidity** | | |
| **Serious** |  |  |
| Ischemic heart disease | 0.006793 | 0.006453 |
| Ischemic stroke | 0.002522 | 0.002107 |
| Hemorrhagic stroke | 0.000765 | 0.00061 |
| Cirrhosis of the liver | 0.000375 | 0.000525 |
| Acute Pancreatitis | 0.000607 | 0.00051 |
| **Temporary** |  |  |
| Lower respiratory infections | 0.006793 | 0.006453 |
| Transport injuries | 0.004343 | 0.003833 |
| Injuries other than from transport | 0.070186 | 0.061976 |
|  |  |  |
| **Mortality** | | |
| **Serious** |  |  |
| Ischemic heart disease | 0.0011518 | 0.0005509 |
| Ischemic stroke | 0.0002234 | 0.0001758 |
| Hemorrhagic stroke | 0.0001265 | 0.0000971 |
| Cirrhosis of the liver | 0.0000298 | 0.0000209 |
| Acute Pancreatitis | 0.0000132 | 0.0000097 |
| **Temporary** |  |  |
| Lower respiratory infections | 0.0001786 | 0.0000972 |
| Transport injuries | 0.0011327 | 0.0005365 |
| Injuries other than from transport | 0.0000635 | 0.0000166 |

**Table A13.** Cost Breakdown of MET and BSCT Interventions per Patient (costs in 2022 $US)

| Category | MET | BSCT | Notes |
| --- | --- | --- | --- |
| Number of sessions | 4 | 5 |  |
| Therapist time per session (hours) | 1.75 | 1.75 | 1h prep + 0.75h session |
| Therapist hourly rate ($) | 25.76 | 25.76 | Based on avg monthly salary of $4,121 |
| Cost per session (therapist time) | $45.08 | $45.08 | 1.75h * $25.76/h |
| Material cost per session | $0.05 | $0.30 | $0.20 / 4 sessions for MET, $1.48 / 5 sessions for BSCT |
| Total cost per session | $45.13 | $45.38 |  |
| Total session cost | $180.52 | $226.90 | Cost per session * Number of sessions |
| Supervision time per therapist (hours) | 36 | 36 | 2h x 3 occasions per semester / 6 semesters |
| Supervisor hourly rate ($) | 27.50 | 27.50 | Based on avg monthly salary of $4,402 |
| General supervision cost | $990.00 | $990.00 | 36h * $27.50/h |
| MI coding cost (MET only) | $84.08 | N/A | Additional for MET |
| Total supervision cost | $1,074.08 | $990.00 |  |
| MET Coding per Manual | $19.78 | N/A | Additional for MET |
| Facility cost | $59.35 | $59.35 |  |
| Overhead cost | $31.65 | $31.65 | Administration, electricity, phone, etc. |
| Total cost per patient ($) | $1,365.38 | $1,307.90 | Sum of all costs |
| Rounded total cost ($) | $1,365 | $1,308 |  |
